# Supplementary material for: Effect of Electron Donating Functional Groups on Corrosion Inhibition of J55 Steel in a Sweet Corrosive Environment: Experimental, Density Functional Theory, and Molecular Dynamic Simulation
Source: Materials (Basel). 2018 Dec 21;12(1):17. doi: 10.3390/ma12010017 (PMC6337217; doi:10.3390/ma12010017)
Supplement: Supplementary file 1 [file materials-12-00017-s001.pdf]

materials

Supplementary Materials: Effect of Electron Donating Functional Groups on Corrosion Inhibition of J55 Steel in a Sweet Corrosive Environment: Experimental, Density Functional Theory, and Molecular Dynamic Simulation

Ambrish Singh, Kashif R. Ansari, Mumtaz A. Quraishi and Hassane Lgaz

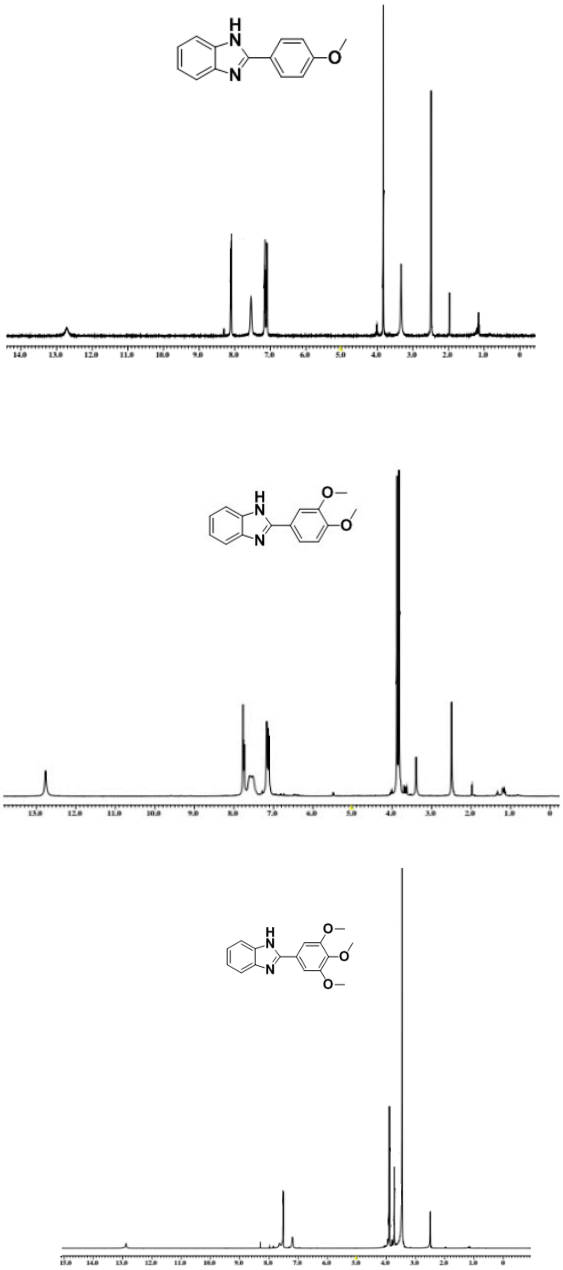

Figure S1. ^{1}H NMR spectra.

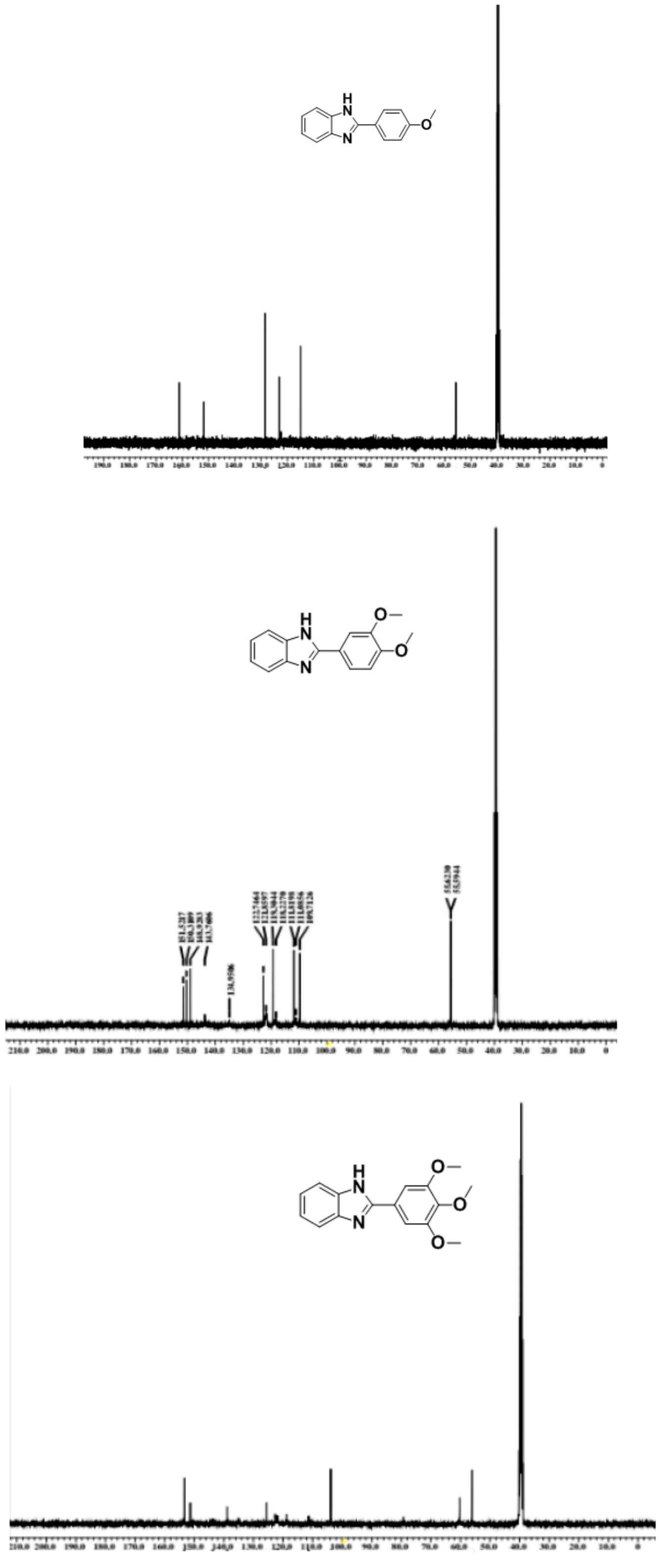

Figure S2. ^{13}C NMR spectra.

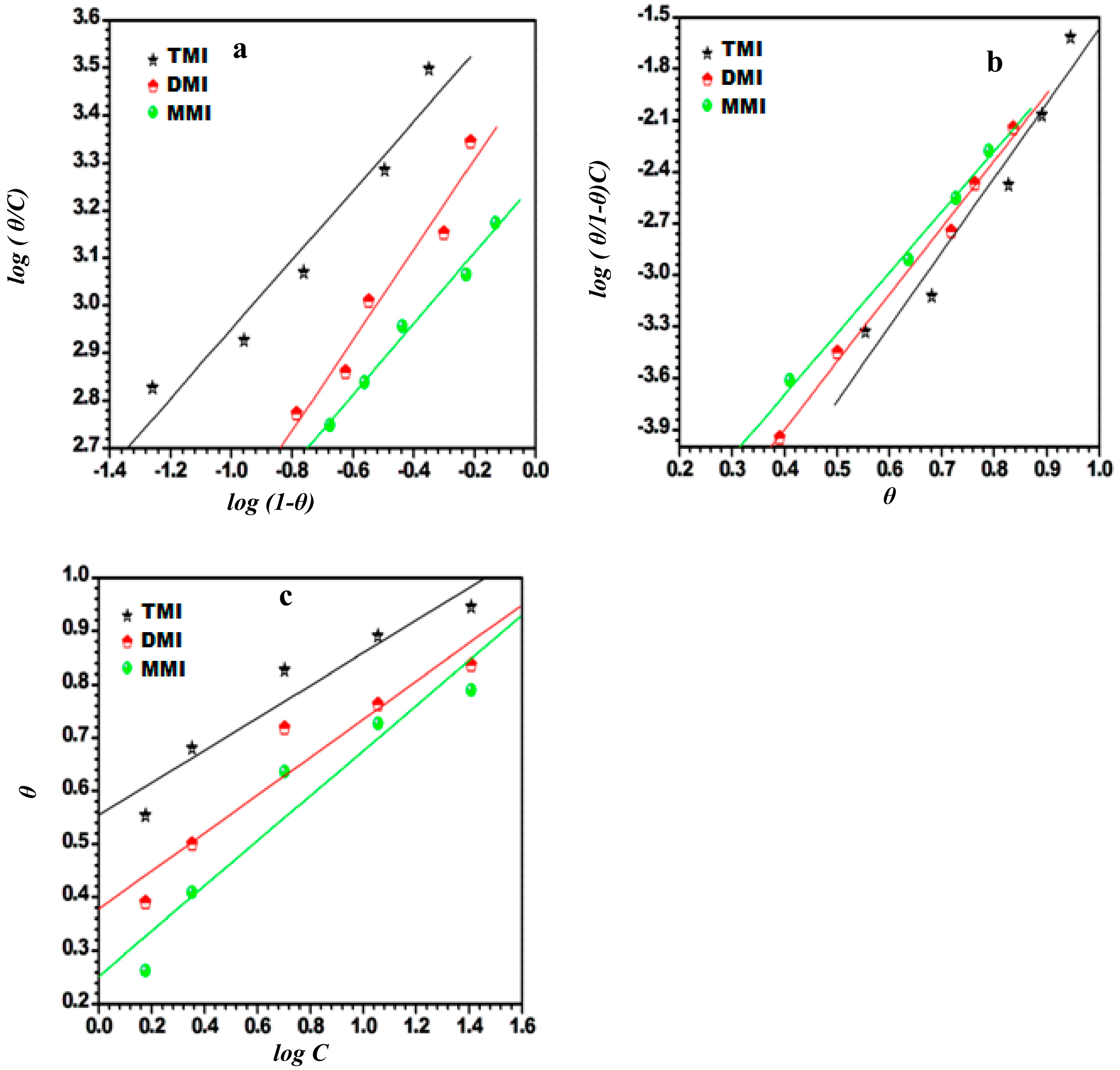

Figure S3. Adsorption isotherm plots: (**a**) Flory-Huggins, (**b**) Frumkin, (**c**) Temkin.

© 2018 by the authors. Licensee MDPI, Basel, Switzerland. This article is an open access article distributed under the terms and conditions of the Creative Commons Attribution (CC BY) license (http://creativecommons.org/licenses/by/4.0/).
